# Association of Screen Time Usage and Physical Activity With Overweight and Obesity Among School-Going Children in Uttar Pradesh

**DOI:** 10.7759/cureus.47690

**Published:** 2023-10-25

**Authors:** Abha Kaul, Neha Bansal, Prakhar Sharma, Satinder Aneja, MP Mahato

**Affiliations:** 1 Pediatrics, School of Medical Sciences and Research, Greater Noida, IND; 2 Pediatrics, Lady Hardinge Medical College, New Delhi, IND

**Keywords:** prevalence, early intervention, physical activity, screen time, childhood obesity

## Abstract

Background

Being overweight during childhood refers to excess weight for a given height, while obesity denotes excess body fat. These conditions stem from surplus calorie intake and insufficient physical activity. Escalating pediatric obesity is linked to modern sedentary lifestyles, marked by increased screen time, reduced exercise, and poor diets. Once believed to be a concern in affluent nations, obesity now affects developing countries like India due to changing eating habits and urbanization. Despite limitations in measurement tools, such as body mass index (BMI) and waist circumference, recognizing sedentary behaviors such as prolonged screen time is pivotal. The rapidly rising prevalence of pediatric obesity has become a major public health concern; therefore, we conducted this study to determine the prevalence and association of screen time usage with being overweight in school-going children (aged 8-15 years).

Methodology

This observational, cross-sectional study was conducted in Greater Noida, Uttar Pradesh over 18 months (January 2019 to June 2020) after obtaining institutional ethical committee approval. Participants were 8 to 15-year-old students from three co-educational secondary schools in the region. Children with motor or developmental disabilities were excluded. Written informed parental consent and school permission were secured. Anthropometric measurements included weight (SECA 874 U scale) and height (SECA213 stadiometer), which were used to calculate BMI. Overweight/obesity status followed the Indian Academy of Pediatrics guidelines. A validated questionnaire assessed screen time, and a validated Physical Activity Questionnaire measured physical activity. Both questionnaires were administered twice to validate data. SPSS version 23.0 (IBM Corp., Armonk, NY, USA) was used for data analyses (descriptive, t-test, analysis of variance (ANOVA) test, and chi-square test). P-values <0.05 were considered significant.

Results

This study involved 604 participants. Among them, 47.7% had a normal BMI, 37.4% were overweight, and 14.9% were obese. Most participants (97.4%) reported screen time of over 60 minutes daily, while 2.6% reported lower screen time. ANOVA revealed significant differences in daily (F = 16.014, p < 0.001) and weekly (F = 16.175, p < 0.001) screen time among BMI categories. Low physical activity was prevalent (97.7%). ANOVA showed significant variations in physical activity scores and durations (p < 0.001), with normal-weight individuals exhibiting higher levels.

Conclusions

The rising prevalence of overweight among children underscores the need for early intervention strategies, emphasizing the importance of reducing screen time and promoting increased physical activity. These measures are critical in addressing the growing challenge of being overweight during childhood and its potential long-term health implications.

## Introduction

The term overweight refers to excess body weight for a particular height, whereas obesity is used to define excess body fat [1]. Overweight and obesity primarily occur due to excess calorie intake, insufficient physical activity, or both. Pediatric obesity has become a major health issue due to changes in lifestyles such as an increase in screen time, a decrease in physical activity, and poor dietary patterns. Childhood obesity is a forerunner of metabolic syndrome, poor physical health, mental disorders, and glucose intolerance.

Obesity and overweight among children were primarily considered diseases of developed countries with high per-capita income [2]. However, developing countries like India are also joining this pool because of the rapid change in food habits and lifestyle. The burden of overweight and obesity is increasing in India and their onset at an early age is a major public health concern [3].

The prevalence of obesity and overweight increased from 16.3% in 2001 to 19.3% among the adolescent population in studies reported after 2010 [4-7]. Urbanization has been identified as a significant contributing factor to the rising prevalence of obesity among children in India. Body mass index (BMI) is conventionally used to define obesity, although it does not measure excess body fat [8]. Although waist circumference is a better indicator, due to a lack of Indian standards, it is not widely used. The National Childhood Obesity Task Force reported waist circumference to be highly sensitive and accurate for assessing central obesity in children, but acknowledged that there are no Indian results available [9].

The easy availability of electronic devices and network services has led to a significant overall increase in screen time. Previous studies focused mostly on television viewing only [10,11]. India has a limited number of studies that have explored the use of screen-based media and its effect on child health. Although time spent watching television has typically been the focus of sedentary behavior studies, other domain-specific sitting behaviors such as using the computer, playing electronic games, reading, talking on the telephone, using social media, and other small-screen recreation also contribute to young people’s sedentary time [10,11].

The increase in screen time needs to be addressed and caregivers need to be educated about early interventions to decrease the burden of overweight and obesity in our population. As the prevalence of pediatric obesity has become a major public health issue, we conducted this study to determine the prevalence and association of screen time usage with overweight and obesity in school-going children (aged 8-15 years).

## Materials and methods

This observational, cross-sectional study was conducted in three different schools located in Greater Noida, Uttar Pradesh during the period of 18 months from January 2019 to June 2020. Children and adolescents of either sex aged 8-15 years were enrolled. Children and adolescents with motor and developmental disabilities were excluded. Permission from the respective school authorities, the principal, parents, and the children was obtained before the enrolment.

The sample size for the study was calculated to be 600 using the formula n = 4pq/(L)². In this equation, the prevalence (p) derived from previous studies was 15%, while the complementary value (q) was set as 85% (100 - p). Additionally, a margin of error (L) equal to 20% of the prevalence was considered.

A semi-structured, pre-tested questionnaire was administered by the investigator to each student with the help of a class representative. In 10% of the students, answers were confirmed by telephonic contact with the parents to check if the answers provided by the children were reliable. These questionnaires were pre-tested by initially establishing face validity in 20 children among a subset of our intended population. Forward translation from the original language, English to the native language Hindi, and backward translation, from Hindi to English, were done, and, subsequently, misunderstandings or unclear words were identified. All versions of the translation were reviewed, and it was determined that the original and translated versions achieved semantic, idiomatic, experimental, and conceptual equivalence. The questionnaires were administered on a particular day which would approximately take 20 minutes, and after two weeks, the same questionnaires were administered to the same subset of the population, and recall bias was checked. These questionnaires were administered first and later the anthropometric measurements were recorded.

Measurements of height, weight, and mid-arm circumference were recorded in the proforma. Anthropometric measurements including body weight nearest to 0.1 kg using SECA 874 U electronic weighing scale and height nearest to 0.1 cm with SECA 213 portable stadiometer were obtained. BMI (kg/m^2^) was calculated using these parameters. BMI was calculated using the following formula: BMI = weight in kilograms divided by height in meters squared. To define overweight and obesity in children aged 5-18 years, the adult equivalent of 23rd and 27th cut-off lines, as presented in BMI charts, were used according to the Indian Academy of Pediatrics (IAP) Growth Monitoring Guidelines for Children from birth to 18 years. These children were then classified according to IAP guidelines [12].

A screen time questionnaire was administered to students in the school in which the self-reporting by the students regarding the screen time usage in the last seven days was recorded. This was a validated questionnaire and was adapted from a study that assessed screen time behavior in Ireland [11]. It included questions regarding time spent watching TV, videos, and DVDs; computers for homework; and the use of mobile phones and tablets for gaming, social media, etc. This questionnaire did not include time spent using computers in school. A total screen time scale variable was also created which combined all screen time variables (television, computer, and video games). A higher score on all variables reflected higher levels of screen time. This data was confirmed by contacting the parents of 10% of the study population as previous studies have shown that the parental estimates of screen time usage were in concordance with the data provided by the children [10].

The Physical Activity Questionnaire (PAQ-C) used in this study is a validated questionnaire that helped assess the duration of physical activity of each student. The questionnaires were administered by the investigator in English and Hindi. The questionnaire had points for each question and once we had a value from 1 to 5 for each of the eight items (items 1 to 8) used in the physical activity composite score, we calculated the mean of the eight items, which resulted in the final PAQ activity summary score. A score of 1 indicated low physical activity, whereas a score of 5 indicated high physical activity [13].

The study data were entered in Microsoft Excel and analyzed using SPSS Software version 23.0 for Windows (IBM Corp., Armonk, NY, USA). Descriptive results were expressed as the mean and SD of various parameters in the different groups. The comparison of continuous variables was assessed by Student’s t-test, multiple groups mean using analysis of variance (ANOVA) test with post hoc using Games-Howell, and categorical variables were assessed using the chi-square test. P-values less than 0.05 were considered significant, and p-values less than 0.001 were considered highly significant.

Ethical considerations

The non-disclosure of the individual’s identity and the information provided was kept in utmost confidentiality and was used only for academic purposes. Institutional Ethical Committee, School of Medical Sciences and Research and Sharda Hospital, Sharda University issued study approval (approval number: SU/SMS&R/76-A/2018/108, dated 27/11/2018).

## Results

In the present study, a total of 604 children were enrolled. All students were attending the private schools of Greater Noida, Uttar Pradesh. These were co-educational, English-medium schools with monthly fees of about 10,000 INR per month. According to government regulations, 10% (60) of the students from economically weaker sections are admitted to these schools. They had large playgrounds, with some having dedicated sports training activities after school hours as well. Children up to class 12 were not allowed to carry cell phones to school.

In our study, the majority of children were in the 12-14-year age group. This study had a preponderance of girls. The socioeconomic status of the participants was determined using the Modified Kuppuswamy scale. The majority of the children belonged to the upper-middle socioeconomic class (328, 54.3%), while others belonged to the upper socioeconomic status (118, 19.5%) and lower-middle socioeconomic status (158, 26.2%). The majority of the students came from an affluent background, with none of the children belonging to lower socioeconomic status. Among the 604 participants, 47.7% (288) were categorized as having a normal BMI, 37.4% (226) were classified as overweight, and 14.9% (90) were obese. Among the 604 participants, 35.1% (212) reported engaging in physical activity for less than 60 minutes per day, while the majority (64.9%, 392) reported being active for more than 60 minutes daily. The majority (97.7%, 590) were classified as having low physical activity based on their PAQ scores, while a small proportion (2.3%, 14) were categorized as having high physical activity levels. A majority of the participants (97.4%, 588) reported screen time exceeding 60 minutes per day, while only a small proportion (2.6%, 16) reported spending less than 60 minutes on screens daily (Table 1).

**Table 1:** Baseline characteristics of the children. BMI: body mass index; PAQ: Physical Activity Questionnaire

Baseline characteristics	Frequency	%
Age (in years)		
9	4	0.7
11	96	15.9
12	146	24.2
13	146	24.2
14	142	23.5
15	70	11.5
Gender		
Male	226	37.4
Female	378	62.6
Religion		
Hindu	588	97.4
Muslim	14	2.3
Kuppuswamy scale		
Upper	118	19.5
Upper middle	328	54.3
Lower middle	158	26.2
BMI category		
Normal	288	47.7
Overweight	226	37.4
Obesity	90	14.9
Total activity per day		
Less than 60 minutes	212	35.1
More than 60 minutes	392	64.9
PAQ score interpretation		
Low physical activity	590	97.7
High physical activity	14	2.3
Screen time per day		
Less than 60 minutes	16	2.6
More than 60 minutes	588	97.4

In our study, the total physical activity minutes per day were recorded for 604 participants, with a minimum of 10.0 minutes, a maximum of 150.0 minutes, a mean of 74.17 minutes, and an SD of 35.40. Similarly, the total physical activity minutes per week were assessed for the same number of participants, ranging from 70.0 to 1,050.0 minutes, with a mean of 519.26 minutes and an SD of 247.78. Screen time per day in minutes was reported by 604 participants, ranging from 50.0 to 480.0 minutes, with an average of 195.49 minutes and an SD of 103.05. Additionally, screen time per week in minutes for the same number of participants ranged from 350.0 to 3,360.0 minutes, with an average of 1,368.47 minutes and an SD of 721.39 (Table 2).

**Table 2:** Mean total physical activity and total screen (minutes per day and per week) among children. SD: standard deviation

Variables	N	Minimum	Maximum	Mean	SD
Total physical activity minutes per day	604	10.0	150.0	74.17	35.40
Total physical activity minutes per week	604	70.0	1,050.0	519.26	247.78
Screen time per day in minutes	604	50.0	480.0	195.49	103.05
Screen time per week in minutes	604	350.0	3,360.0	1,368.47	721.39

In our study, statistically significant differences were observed among the three BMI categories for physical activity score (F = 17.351, p < 0.001), total physical activity minutes per day (F = 26.126, p < 0.001), and total physical activity minutes per week (F = 30.372, p < 0.001). Post-hoc analysis using the Games-Howell method further revealed significant group differences. Regarding physical activity scores, normal-weight individuals had a significantly higher mean score compared to overweight and obese participants (p < 0.001). Likewise, for both total physical activity per day and per week, those with normal BMI exhibited significantly higher mean values compared to their overweight and obese counterparts (p < 0.001). Significant differences were observed among the three BMI categories in terms of both screen time per day (F = 16.014, p < 0.001) and screen time per week (F = 16.175, p < 0.001). Post-hoc analysis using the Games-Howell method revealed specific group differences. Regarding screen time per day, individuals classified as overweight or obese had significantly higher mean values compared to those with a normal BMI (p < 0.001). Similarly, for screen time per week, overweight and obese participants displayed significantly greater mean values compared to their normal-weight counterparts (p < 0.001) (Table 3).

**Table 3:** Comparison of mean physical activity score, total physical activity, and screen time (per day and per week) with BMI category among children. BMI: body mass index; ^a^: normal BMI; ^b^: overweight; ^c^: obesity; *: p-value <0.001 was statistically significant; #: p-value <0.05 was statistically significant.

Variables	BMI category			
	Normal (a)	Overweight (b)	Obesity (c)	P-value
	Mean ± SD	Mean ± SD	Mean ± SD	
Physical activity score	2.8 ± 0.9*^b,c^	2.7 ± 0.5*^a,c^	2.5 ± 0.5*^a,b^	0.001^#^
Total physical activity minutes per day	82.0 ± 38.3*^b,c^	73.9 ± 31.7*^a,c^	49.8 ± 20.6*^a,b^	0.001^#^
Total physical activity minutes per week	574.2 ± 267.7*^b,c^	517.3 ± 222.1*^a,c^	348.4 ± 144.3*^a,b^	0.001^#^
Screen time per day in minutes	173.0 ± 91.5*^b,c^	200.9 ± 102*^a,c^	254.0 ± 116.2*^a,b^	0.001^#^
Screen time per week in minutes	1,210.9 ± 640.2*^b,c^	1,406.2 ± 713.9*^a,c^	1,778.0 ± 813.7*^a,b^	0.001^#^

Multiple regression was used to predict the BMI of children from physical activity, screen time, age, and gender. The multivariate analysis model predicted/explained 28.0% variance in the BMI of children. The variables statistically predicted the BMI (F(4,599) = 59.133, p < 0.001, R^2^ = 2.83). All four variables added statistically significantly to the prediction (p < 0.05). The general form of the equation to predict the BMI from physical activity, screen time, age and gender was BMI = 11.071 - (0.005 × physical activity) - (0.001 × screen time) + (0.064 × age) + (0.566 × gender). Overall, the model predicted BMI variation by 28%. In the model, physical activity contributed 36.0%, screen time 20.0%, age 30.0%, and gender 8.0% toward the increase in the BMI of children (Table 4).

**Table 4:** Multivariate logistic regression to determine significant contributors for the variation in BMI. SE: standard error; ^a^: dependent variable body mass index (BMI); *: p-value <0.05 was considered significant.

Variables	Coefficient of beta	SE	P-value	Lower bound	Upper bound
(Constant)	11.071	1.292	0.000*	8.534	13.607
Total physical activity in minutes per week^a^	-0.005	0.000	0.000*	-0.006	-0.004
Screen time per week in minutes per week^a^	0.001	0.000	0.000*	0.001	0.001
Age in months^a^	0.064	0.007	0.000*	0.049	0.078
Gender^a^	0.566	0.238	0.018*	0.099	1.034

## Discussion

Overall, the prevalence of overweight and obesity among these children was 52.3% (316) (37.4% (226) and 14.9% (90), respectively) according to IAP recommendations. The prevalence of overweight and obesity is much higher than that reported in the Comprehensive National Nutrition Survey (CNNS). According to the CNNS 2018 report, 1.6% of the children aged 5-9 years were overweight and 0.5% were obese, whereas among children aged 10-19 years, the prevalence of overweight was 2.3% and obesity was 0.5% in Uttar Pradesh [14]. The overall prevalence of overweight was 9.9% (89) and obesity was 4.8% (43) in a study by Kotian et al. (2010) in Karnataka among 900 adolescents aged 12 to 15 years [15]. The prevalence of overweight and obesity was 9.8% (65) and 4.8% (32) among 660 adolescents in Aligarh, respectively, in a study by Nawab et al. [16]. In a study from Chennai by Sundar et al., the prevalence of obesity and overweight among urban school children aged 13-17 years was 11.5% (46) and 3.7% (15), respectively [17]. The prevalence of overweight and obesity in a survey in South India was found to be 18.7% (163) and 5.8% (52), respectively, among adolescents aged 10-19 years [18]. In concordance with our study, Haq et al. recorded a lower prevalence of overweight and obese children on application of the WHO growth standards in comparison with the IAP and International Obesity Task Force [19].

In this study, there was a highly significant association between overweight and obesity with the total activity of a child per day. Overall, 68.8% (62) of obese children had a total activity of fewer than 60 minutes per day, whereas 32.7% (74) of overweight and only 26.3% (76) of children with normal weight had a total activity of fewer than 60 minutes per day. These findings were statistically highly significant (p < 0.001). Our study also showed a significant correlation between total physical activity and BMI. Similar to the above findings, the previous studies documented that regular physical activity is inversely related to obesity, and the percentage of overweight and obesity was high in those who did not exercise regularly compared to those who did exercise regularly and in those who exercised for fewer than two hours a day [20]. In a study by George et al., conducted among 485 school children in New Delhi, the prevalence of overweight and obesity was 9.5% (46) and 11.5% (56), respectively, and statistically 43.8% (212) of children were physically active for at least one hour a day on all seven days of the previous week [21]. Just 29% (479) of adolescents were engaged in daily moderate-to-vigorous exercise in a study of urban adolescents in Kolkata [22]. The study by Hazzaa et al. described that other lifestyle factors were associated with obesity and weight gain among children [23]. According to the WHO guidelines, children and youth aged 5-17 years should perform at least 60 minutes of daily moderate-to-intense physical activity. The WHO also recommends that much of the daily physical activity should be aerobics and that intense intensity exercises, including those that strengthen muscles and bones, should be implemented at least three days a week [24,25].

In our study, 97.4% (590) of the population had screen time usage of more than 60 minutes per day, and among the children who were overweight and obese, all were found to have screen time of more than 60 minutes per day. There was a highly significant association between screen time usage and the occurrence of overweight and obesity among these children (p < 0.001). Our study also showed a significant correlation between the total screen time usage with the BMI of the children. Approximately 68% (37) of adolescents in a study on screen time assessment among adolescents in Delhi reported screen time of more than two hours, with the mean duration of screen time usage among the adolescents being 3.8 hours [26]. In a survey of 1,063 Chinese students aged 8-19 years, extended screen time of about two hours was observed in 14.7% (156) of boys and 8.9% (95) of girls [27]. In a computer-based analysis of teenage behaviors in Bangladesh, adolescents were found to have screen time greater than two hours a day [28]. The American Academy of Pediatrics Guidelines (2016) suggests that clear restrictions be imposed on the time spent using media and media forms in children six years of age and older to ensure that media do not take the place of sufficient sleep, physical activity, and other health-critical behaviors [29].

Limitations

The study is subject to some limitations. First, the data on physical activity and screen time were collected through self-reporting, introducing the potential limitations associated with self-reporting, such as recall bias or social desirability bias, where students may underreport screen time or provide answers they perceive as more socially acceptable. Additionally, the sample predominantly comprised individuals from urban and higher socioeconomic backgrounds, potentially affecting the study’s generalizability to the broader population.

## Conclusions

The need of the hour to curb obesity among children and adolescents is awareness of this new-age issue and measures to reduce screen time and encourage a physically active lifestyle. Interventions need to be implemented at a very early age as we have seen that the problem of overweight and obesity starts setting in at a very early age of around 12 to 13 years. We need to take steps even before this age to avoid the burden of this disease in adolescence and adulthood. Involvement of children in physical activity should be taken care of at the school and children should spend a major portion of the day at school. Strategies to limit screen time usage need to be enforced to promote physical activity which would ultimately increase metabolic activity and help reduce the burden of the disease. Active involvement of parents with their children would help us achieve better results.
